# Diagnosis of occlusal dysesthesia utilizing prefrontal hemodynamic activity with slight occlusal interference

**DOI:** 10.1002/cre2.32

**Published:** 2016-06-14

**Authors:** Yumie Ono, Yu Ishikawa, Motohiro Munakata, Tomoaki Shibuya, Atsushi Shimada, Hideo Miyachi, Hiroyuki Wake, Katsushi Tamaki

**Affiliations:** ^1^ Health Science and Medical Engineering Laboratory, Department of Electronics and Bioinformatics, School of Science and Technology Meiji University Kawasaki Japan; ^2^ Department of Oral Implantology Kanagawa Dental University Hospital Yokosuka Japan; ^3^ Department of Prosthodontic Dentistry for Function of TMJ and Occlusion Kanagawa Dental University Yokosuka Japan; ^4^ Department of Special Denture and Occlusion & Liaison Kanagawa Dental University Hospital Yokosuka Japan; ^5^ Department of Psychiatry Kitasato University School of Medicine Sagamihara Japan

**Keywords:** diagnostic criteria, near‐infrared spectroscopy, occlusal discomfort syndrome, occlusal dysesthesia, phantom bite syndrome, somatoform disorder

## Abstract

Clinical diagnosis of occlusal dysesthesia (OD), also referred to as phantom bite syndrome, is currently based on the absence of objective occlusal discrepancy despite the persistent complaint of uncomfortable bite sensation. We previously demonstrated that the subjective feeling of occlusal discomfort generated by artificial occlusal interference can be objectively evaluated using prefrontal hemodynamic activity in young healthy individuals. The aim of this study was to investigate whether dental patients with and without OD show distinct prefrontal activity during grinding behavior with an occlusal interference. Six dental patients with OD (OD group) and eight patients without OD (control group) grinded piled occlusal strips placed between their first molars and reported their perception and discomfort thresholds during continuous monitoring of prefrontal hemodynamic activity with a portable functional near‐infrared spectroscopy. Although patients without OD showed the typical hemodynamic pattern of increased oxyhemoglobin and reduced deoxyhemoglobin (HHb) concentration, those with OD showed persistent incremental increases of HHb concentration that began at the loading of occlusal strips on their molars before they executed grinding. The intensities of the task‐related HHb activities showed statistically significant differences between OD and control groups, particularly at channel 3, arranged over the left frontal pole cortex. When the discrimination criterion was set using the intensity values of channel 3 from both groups, the overall accuracy of the OD discrimination was 92.9%. Although physiological interpretation has yet to be elucidated, the task‐related response of an increase in HHb may be a useful neuronal signature to characterize dental patients with OD.

## Introduction

Occlusal dysesthesia (OD) (Clark & Simmons, 2003), also referred to as phantom bite syndrome (Marbach, 1976) or a narrow sense of occlusal discomfort syndrome (Tamaki et al., 2016), is a symptom characterized by “a persistent complaint of uncomfortable bite sensation with no obvious occlusal discrepancy” (Hara et al., 2012; Melis & Zawawi, 2015). Currently, OD is accepted as a derived form of the somatoform disorder in which the symptom is mostly expressed in the oral area, and history of dental occlusal procedures may trigger the symptom (Hara et al., 2012; Melis & Zawawi, 2015). Dental therapy, such as occlusal adjustment, fails to relieve discomfort in OD patients; therefore, it is necessary to diagnose patients with OD from other dental patients early to avoid unnecessary dental treatment and to improve patient quality of life.

We have previously shown that the intensity of prefrontal hemodynamic activity accurately reflects the subjective intensity of simulated occlusal discomfort in healthy individuals (Ono et al., 2015). In the present study, we adopt a similar paradigm of the combined use of simulated bite rise and functional near‐infrared spectroscopy (fNIRS) to investigate whether prefrontal hemodynamic activity under slight occlusal interference differs between OD patients and age‐matched and gender‐matched control dental patients without OD. With its unique feature of tolerance to body movement, fNIRS has been used to investigate regional cortical activity related to jaw movement as a reliable functional brain imaging modality in the field of dentistry (Shibusawa et al., 2009; Narita et al., 2009; Iida et al., 2012). The current hypothesis is that the hemodynamic response from the frontal pole cortex (FPC), the most anterior part of the prefrontal cortex, may show a distinct response pattern in OD patients. Although the primary role of the FPC in a variety of human cognitive functions is still debated, Christoff and Gabrieli (2000) suggested that an activation of FPC indicates “monitoring and manipulation of internally represented information” that has been previously or originally experienced, to assist the cognitive processing of “externally generated,” ongoing information in the dorsolateral prefrontal cortex (DLPFC). Participants in the current study were instructed to judge if the thickness of their raised bite was at their perception or discomfort threshold in order to compare altered proprioceptive occlusal perceptions with intrinsic occlusal perceptions. Consequently, the activation of FPC and the concomitant increase of blood flow signal in fNIRS are expected in this paradigm. We aimed to determine whether the FPC area of OD patients, whose self‐body image of their dental occlusion had been altered, exhibited a hemodynamic response pattern required to judge occlusal vertical dimension. An additional aim was to investigate the utilization of different hemodynamic response patterns between patients with and without OD to classify these two patient groups given their distinct hemodynamic time courses.

## Materials and Methods

### Participants

Six patients with OD (one man, five women; mean age ± standard deviation [SD] 49.5 ± 7.5 years; OD group) and eight age‐matched dental patients without OD (all women, mean age ± SD 59.3 ± 8.0 years; control group) participated in the experiment. Mean patient age and gender distribution conformed to those in a previous report of 37 cases (51.7 ± 10.6 years, male/female ratio: 1/5.1) (Hara et al., 2012). OD was diagnosed by one psychiatrist and three dental clinicians based on criteria proposed by Melis and Zawawi (2015). Briefly, the criteria for OD patients comprised complaint of uncomfortable bite sensation for at least 6 months in the absence of dental occlusal discrepancies or disproportional to the complaint. Control group participants were patients who regularly visited the Kanagawa Dental University Hospital for prosthodontic apparatus maintenance. Inclusion criteria for control group comprised no complaint of uncomfortable bite sensation for at least 6 months and remaining natural mandibular first molar and its opposing teeth of the habitual chewing side. Clinicians interviewed all participants and assessed their stomatognathic function upon arrival at the clinic to ensure that they were free from psychiatric symptoms and any objective stomatognathic symptoms that required treatment such as caries, periodontal disease, and temporomandibular dysfunction. Exclusion criteria therefore comprised (i) existence of prosthetic apparatus (bridge, removable denture, and/or tooth implant) and (ii) caries and/or severe periodontal disease around the mandibular first molar and its opposing teeth of the habitual chewing side. Occlusal contact between the tested teeth was confirmed in all participants with 12‐µm‐thick occlusal paper. This study followed the protocol for the use of human participants and was approved by the Ethics Committee of the Kanagawa Dental University Hospital (approval no. 214). All participants provided written informed consent after full explanation of the experiment was provided.

### Functional near‐infrared spectroscopy data acquisition

**Figure 1:**
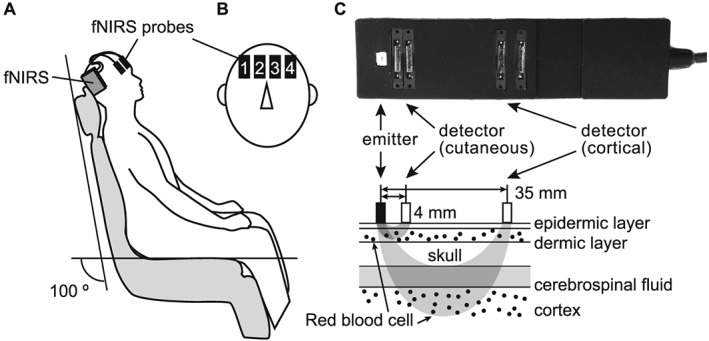
Schematic image of experimental setup. (A) Portable functional near‐infrared spectroscopy (fNIRS) arrangement on a participant sitting in a dental chair. (B) Four optical probes (channels 1–4) are attached on the forehead of the participant. (C) Magnified image of a single probe (photograph on the top) containing one emitter and a pair of detectors for simultaneous measurements of cutaneous and cortical hemodynamic responses, respectively.

Participants comfortably sat in a dental chair at reclining angle of 100° (Fig. 1A). An investigator (M. Munakata or K. Tamaki) inserted a 12‐µm‐thick metal strip (ARTUS; Englewood, NJ) on the occlusal surface of the mandibular first molar of the habitual chewing side and verbally instructed the timing for grinding motion. A block design comprising 10 s of holding the metal strip in the mouth, 15 s of gentle grinding, and 30 s of rest was adopted. Participants closed their jaw with their least occlusal force to hold the metal strip with their maxilla and mandibular molar teeth to perform grinding. Four‐channel wireless fNIRS probes (Hb13; Astem Co. Ltd, Kanagawa, Japan) were positioned over the prefrontal cortices. Probes were attached above Fp1, Fp2, F7, and F8 according to the international 10–10 system, corresponding to bilateral FPCs and inferior frontal gyri ((Okamoto et al., 2004); Fig. 1B). Each optical probe comprised a single emitter and two detectors with different emitter‐detector intervals (4 and 35 mm; Fig. 1C) to simultaneously record the skin and cortical blood flow at a sampling rate of 2 Hz. Measurements were repeated by increasing the thickness of metal strips (12 µm per each strip) through stacking from 0 µm (only the strip holder was inserted into the mouth) until the thickness at which the participant perceived occlusal discomfort was reached. Participants communicated their perception and discomfort thresholds to the investigator by hand signs after the rest period of each trial.

### Data analysis

Previous studies have shown that the occlusal vertical dimensions at perception and discomfort thresholds vary among participants (Ono et al., 2015). Therefore, we first compared the time course of fNIRS responses between the OD and control group during (i) grinding without metal strip, and grinding metal strips with thicknesses at their (ii) individual perception and (iii) discomfort threshold. The fNIRS waveforms of each trial were separately normalized with SD values during 5 sec before initiating holding motion and were baseline corrected at the onset of holding motion to enable comparison between participants. The peak amplitudes of the normalized and baseline‐corrected fNIRS responses were compared among the three conditions to investigate the difference in fNIRS responses related to the grinding with different occlusal perceptions. The aforementioned analysis demonstrated that the time course of these waveforms were almost identical in a single participant group at these three conditions, which were almost equal or below the minimal discomfort threshold. Therefore, fNIRS waveforms were averaged across all trials for each participant to improve the data's signal‐to‐noise ratio. The averaged, normalized, and baseline‐corrected data were used for further analysis.

To confirm that the observed fNIRS response mainly originating from the cortex, we measured the signals obtained from two different interprobe intervals. Because of the path length of the near‐infrared light, fNIRS signals obtained from an interprobe interval of 4 mm can be considered as signals mostly reflecting skin blood flow, while those obtained from an interprobe interval of 35 mm are those mainly reflecting cerebral blood flow with little skin blood flow contamination (Fig. 1C).

We also performed a regression analysis of the averaged, normalized, and baseline‐corrected waveforms at a selected channel to determine the task‐dependent activity pattern intensity. A generalized linear model using the hemodynamic response function (HRF; (Friston et al., 1994)) was applied accordingly,

$$y\left(t\right)=a_{1}HRF\left(t\right)+a_{2}t+b+\varepsilon \text{,}$$

where *a*_1_, *a*_2_ denote HRF and time‐course trend coefficients and *b* and *ε* denote baseline and error, respectively. The coefficient *a*_1_ corresponding to the HRF was considered the intensity of the task‐dependent hemodynamic response as previously described (Ye et al., 2009; Ono et al., 2015).

Using the coefficient values of the individual fNIRS signal at the specific channel, we further developed the classifier that could best discriminate the patients with and without OD. In brief, the linear discrimination analysis algorithm (Krzanowski, 1988) was applied to the coefficient values of both groups to determine the discrimination threshold. The mean accuracy of the classifier was determined using the leave‐one‐out cross‐validation algorithm. We also calculated the sensitivity, specificity, positive predictive value, and negative predictive value of the classifier. All analytical procedures were performed by our in‐house developed programs using MATLAB (Natick, MA).

### Statistical analysis

Occlusal vertical dimensions at perception and discomfort thresholds were compared between groups using two‐sample *t*‐test, after the Shapiro–Wilk test confirmed the normality of the data. The peak amplitudes of the grinding‐related fNIRS responses among different occlusal vertical dimensions that were compared using one‐way repeated measures analysis of variance after the Shapiro–Wilk test confirmed the normality of the data. We confirmed the significance of linear regression relationships between the fNIRS waveforms and the predictor responses in all regression models tested using an *F*‐test simultaneously calculated with the regression analysis in MATLAB. Mean coefficient amplitudes were compared between groups using the *t*‐test after data normality was confirmed by the Shapiro–Wilk test.

## Results

### Perception and discomfort thresholds

Control and OD groups showed comparable occlusal vertical dimensions at perception and discomfort thresholds. Discrimination thresholds were 40.5 ± 6.0 and 32.0 ± 8.0 µm in control and OD groups (*P* = 0.401), and discomfort thresholds were 79.5 ± 9.6 and 76.0 ± 19.5 µm in control and OD groups, respectively (*P* = 0.865). There were no statistically significant differences in mean discrimination and discomfort thresholds between participant groups.

### Task‐related increase of deoxyhemoglobin response in OD patients

**Figure 2:**
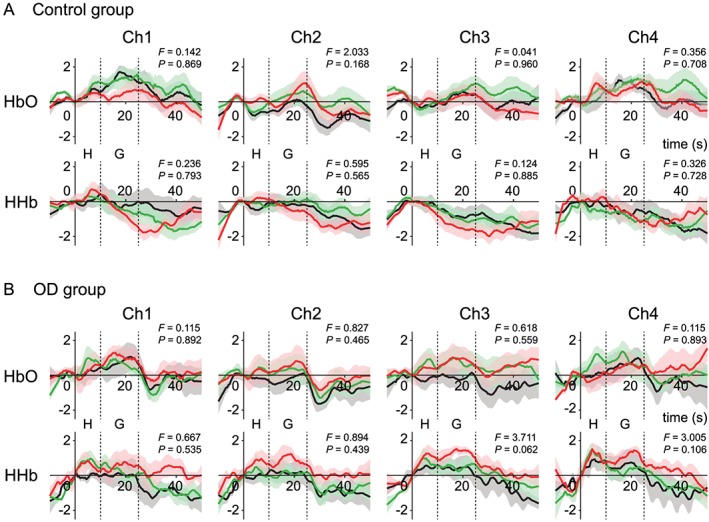
Normalized oxyhemoglobin (HbO) and deoxyhemoglobin (HHb) waveforms during the grinding task with different occlusal vertical dimension of no bite rise (black), perception threshold (green), and discomfort threshold (red) in control group (A) and occlusal dysesthesia (OD) group (B). Shaded areas indicate standard error of mean. Time zero was set at the loading of a strip holder into the mouth, and participants were instructed to maintain a 10‐sec mandible rest position (indicated as “H”: hold). Following the hold period, participants performed a gentle grinding for 15 sec (indicated as “G”: grind). *F* and *P* values obtained from one‐way repeated measures analysis of variance of the peak amplitudes among conditions were also provided.

**Figure 3:**
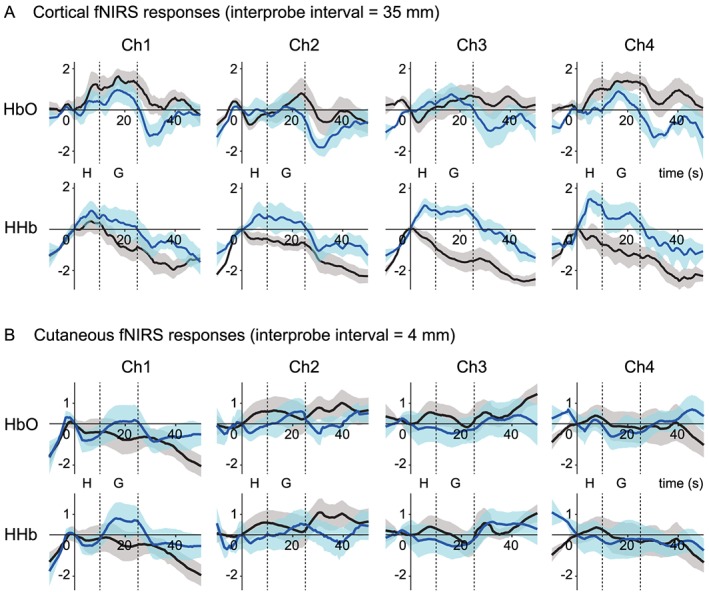
Normalized and averaged oxyhemoglobin (HbO) and deoxyhemoglobin (HHb) waveforms in control group (black) and occlusal dysesthesia (OD) group (blue) at interprobe intervals of 35 (A) and 4 mm (B). Shaded areas indicate standard error of mean. Time zero was set at the loading of a strip holder into the mouth, and participants were instructed to maintain a 10‐sec mandible rest position (indicated as “H”: hold). Following the hold period, participants performed a gentle grinding for 15 sec (indicated as “G”: grind).

**Figure 4:**
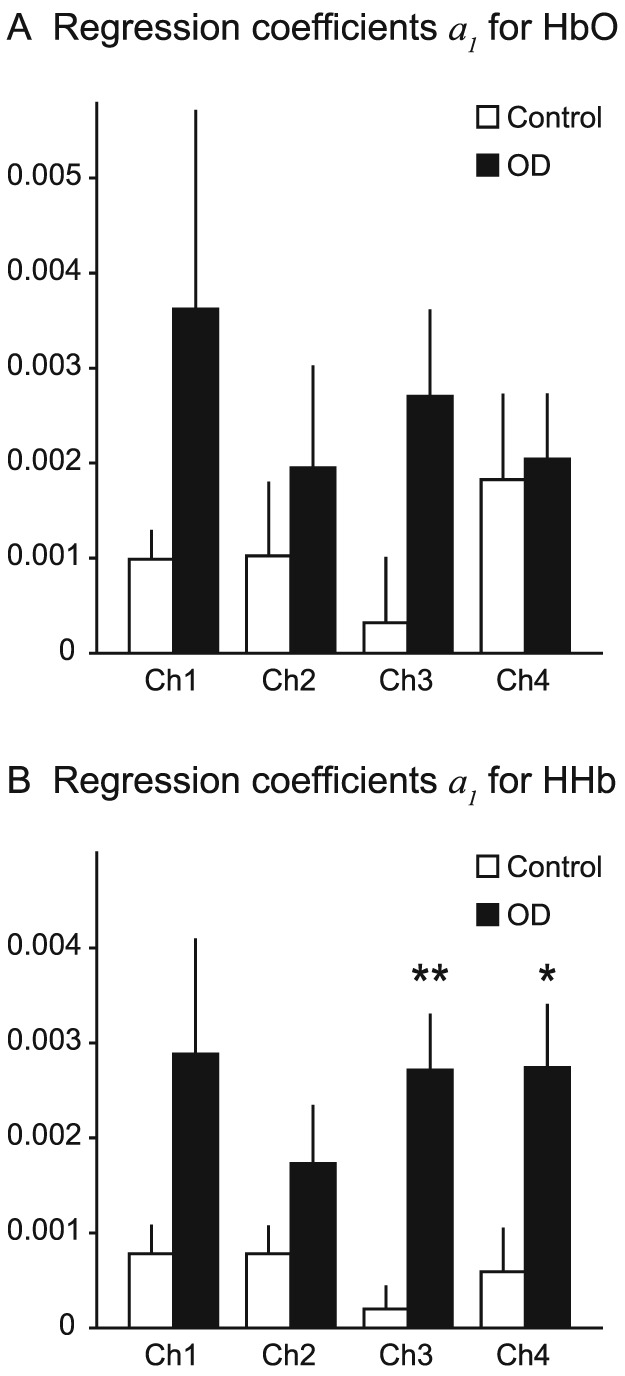
Task‐dependent hemodynamic response intensity comparisons between control and occlusal dysesthesia (OD) groups. Asterisks indicate statistically significant increase of the task‐dependent deoxyhemoglobin response in OD group compared with control (two‐sample *t*‐test, **P = 0.001 and *0.021 at channel (Ch) 3 and Ch 4, respectively).

The time course of the cortical oxyhemoglobin (HbO) and deoxyhemoglobin (HHb) concentration change responses in conditions without bite rise, with occlusal vertical dimension at perception, and with that at discomfort threshold were shown in Figure 2. Although there was a tendency of augmented hemodynamic response found at discomfort threshold compared with the other conditions, neither participant group showed statistically significant differences in the peak amplitudes of hemodynamic responses among conditions (detailed results of the statistical analysis were shown in Fig. 2). Time courses of HbO and HHb concentration change responses averaged across all trials at four cortical (35 mm), and cutaneous (4 mm) fNIRS channels are presented in Figure 3. Hemodynamic response patterns of cutaneous HbO and HHb signals were almost identical (Fig. 3B); however, the cortical HbO and, in particular, the HHb signals showed contrasting pattern between groups. The hemodynamic response of the control group comprised increasing HbO and decreasing HHb. There was little or no increase in HbO signals in the OD group; however, there was a strong task‐dependent increase of HHb, particularly at the channels on the left hemisphere (channels 3 and 4). Furthermore, the increase in HHb signals began from the time around 0 in all channels (Fig. 3A), before the actual grinding is performed. Therefore, the duration of cortical activity in a basal function for the regression analysis *HRF*(*t*) was defined from the loading of occlusal strips (*t* = 0 sec; Fig. 3) until the end of grinding period (*t* = 25 sec; Fig. 3). Regression analyses of cortical HbO and HHb responses further confirmed the significantly larger task‐dependent HHb response in the OD group at channels 3 (*P* = 0.001) and 4 (*P* = 0.021, Fig. 4).

### Classification of occlusal dysesthesia patients depending on prefrontal deoxyhemoglobin signals

We calculated threshold values to discriminate the occurrence of OD using the regression coefficient values of channel 3, showing the highest statistical significance in group comparison. After determining the threshold value in each test and training data sets in the leave‐one‐out cross‐validation, the mean classification accuracy was 92.9%. The overall sensitivity, specificity, positive predictive value, and negative predictive values were 83.3%, 100%, 100%, and 88.9%, respectively.

## Discussion

We measured prefrontal hemodynamic responses during grinding behavior with slight occlusal interference in dental patients with and without OD. Patients with OD showed a task‐related increase in HHb; however, those without OD showed a task‐related reduction in HHb. This suggests that prefrontal hemodynamic responses may be a potential marker for detecting patients with OD. In addition to high discrimination accuracy of OD patients in the regression analysis of HHb responses, this examination requires minimal stress to the patient, because the thickness of the occlusal interference does not exceed the individual discomfort threshold and the portable fNIRS system enables the patient to remain in the dental chair during examination. In addition to the previous studies (Ono et al., 2015; Shibusawa et al., 2009; Narita et al., 2009; Iida et al., 2012) that utilized fNIRS under various jaw movements to understand the cerebral representation of the periodontal sensory input, our results further encourage the use of fNIRS in the clinical dentistry as a quantitative, non‐invasive, and low‐cost diagnostic tool for patients with OD.

We observed a distinct pattern of HbO and HHb responses during the grinding task between patients with and without OD. Corresponding to our previous fNIRS study applying a similar bite rise paradigm to healthy young adult volunteers (Ono et al., 2015), hemodynamic responses in control participants comprised increased HbO and reduced HHb activities. This indicates blood flow increase and the activation of FPC (Scholkmann et al., 2013), suggesting that FPC works to recall and manipulate the patient's self‐oral image to assist the comparison process in the DLPFC (Christoff & Gabrieli, 2000). In contrast, participants with OD showed vague, fluctuated HbO response and a clear, box‐car‐shaped HHb response. The HHb response began at the loading of occlusal strips on molars before grinding was executed (*t* = 0 s) suggesting that this response is associated with top‐down cognitive control, preparing for the upcoming examination of occlusal vertical dimension, as opposed to bottom‐up perception processing based on ongoing motor/proprioceptive afferent information. The potential contamination of the motion artifact and the autonomic change in the cutaneous blood flow can be excluded because cortical HbO and HHb behaved differently, and the time course of the cutaneous blood flow response differed from those of the cortical. Alongside the fact that the HHb responses are less affected by systemic responses, such as breathing cycles and blood pressure changes (Kirilina et al., 2012), we consider that the HHb signal increase in OD patients indicates reduced blood flow in the FPC and cerebral blood flow reallocation to the other part of the cortex. This may imply the suppression of FPC activity during discrimination of occlusal vertical dimension that may account for the variability in an individual's inappropriate occlusal vertical dimension claims due to the suppressed ability of maintaining self‐oral image. This is also supported by the tendency for larger discomfort threshold variance in patients with OD than in controls, although the discrimination threshold mean and variance between these two groups were comparable.

The time course of the hemodynamic responses at discomfort threshold tended to show larger amplitudes compared with those in the other conditions in both participant groups, although the peak amplitudes were not statistically different among the conditions (Fig. 2). The discrepancy in the different subjective sense of occlusion and the comparable prefrontal blood flow responses may arise from a lack of the statistical power to differentiate the minor perceptual differences among the conditions, which were almost equal or below the minimal discomfort threshold. However, we could observe distinct differences between participant groups, even from the single‐trial fNIRS signals such as decreasing and increasing patterns of HHb responses between control and OD groups, respectively, showing the usefulness of the fNIRS examination under the proposed grinding paradigm for characterizing patients with and without OD.

In addition to the averaged hemodynamic responses obtained through all bite rise conditions, we also examined discrimination accuracy using regression coefficient values from hemodynamic responses in the individual trial at no bite rise, discrimination threshold, and discomfort threshold. The best discrimination accuracy was obtained when we utilized the averaged hemodynamic responses. This suggests that averaging responses may be useful to suppress artifacts that are not time‐locked with the grinding task.

There were several limitations to the current study. First, the sample size was small because of the relatively rare occurrence of OD. Accumulating more cases could help determine the general diagnostic criteria of OD via intensity of task‐dependent HHb responses. Second, measured cortical areas were limited to the anterior prefrontal region. The portable fNIRS system utilizes light‐emitting diodes as a light source, and therefore, we were unable to detect signals from cortical areas covered with hair, such as somatosensory cortices and DLPFCs, because of the absorption of near‐infrared light by black hair and hair roots in the Asian participants. Whole‐head fNIRS imaging using a system equipped with high‐power laser diodes as a light source should be utilized to investigate whether the increased HHb response in the FPC is associated with reallocation of blood flow. However, the FPC activity well characterized dental patients with and without OD and is therefore potentially useful for OD diagnosis. The comparable perception and discomfort thresholds between patients with and without OD found in the current study support a previous report (Baba et al., 2005) that indicated the comparative ability of oral sensory perception between OD patients and healthy controls. Our results suggest that the orofacial area of the somatosensory cortices, which receives primary sensory information from peripheral nerves to initiate oral sensory processes in the cerebrum (Trulsson et al., 2010), may be intact in patients with OD and that the altered occlusal perception in the patients with OD may arise from the difference in the cortical activity in the higher cognitive‐processing centers such as the prefrontal cortices. Further comparison of the somatosensory and prefrontal hemodynamic activities with grinding behavior in patients with and without OD would be necessary to elucidate the underlying neural mechanism of OD, although it is beyond the scope of the current study.

In conclusion, we propose a regression analysis of fNIRS signals during grinding behavior to diagnose OD. Our results suggest that HHb response coefficients from the left FPC are significantly different between patients with and without OD and are therefore potentially useful to discriminate the occurrence of OD.

## Conflict of Interest

None declared.
